# 3-(Benzothia­zol-2-yl)-3-(prop-2-yn­yl)hex-5-yn-2-one

**DOI:** 10.1107/S1600536810009293

**Published:** 2010-03-17

**Authors:** Yamna Baryala, Abdelfettah Zerzouf, Moussa Salem, El Mokhtar Essassi, Lahcen El Ammari

**Affiliations:** a Laboratoire de Chimie Organique et Etudes Physico-chimique, ENS Takaddoum, Rabat, Morocco; b Laboratoire de Chimie Organique Hétérocyclique, Pôle de Compétences, Pharmacochimie, Faculté des Sciences, Université Mohammed V-Agdal, Av. Ibn Battouta, BP 1014, Rabat, Morocco; c Laboratoire de Chimie du Solide Appliquée, Faculté des Sciences, Université Mohammed V-Agdal, Avenue Ibn Battouta, BP 1014, Rabat, Morocco

## Abstract

The title compound, C_16_H_13_NOS, was prepared by alkyl­ation of 1-(benzothia­zol-2-yl)propan-2-one with propargyl bromide. The asymmetric unit contains two mol­ecules that are crystallographically independent but linked to each other by non-classical C—H⋯O hydrogen bonds, building up a dimeric substructure. The benzothia­zole rings are essentially planar with maximum deviations of 0.005 (1) and 0.007 (2) Å for the N atoms. Although the two mol­ecules have similar bond distances and angles, they slightly differ in the orientation of the benzothia­zole ring with respect to the two propargyl groups and the acetonyl unit . In the crystal, inter­molecular C—H⋯O inter­actions link the dimeric subunits into a two-dimensional array in the *bc* plane.

## Related literature

For background to the applications of benzothia­zoles in the chemical industry, see: Bradshaw *et al.* (2002 ▶); Delmas *et al.* (2002 ▶); Hutchinson *et al.* (2002 ▶). For the pharmacological activity of benzothia­zole derivatives, see: Repiĉ *et al.* (2001 ▶); Schwartz *et al.* (1992 ▶).
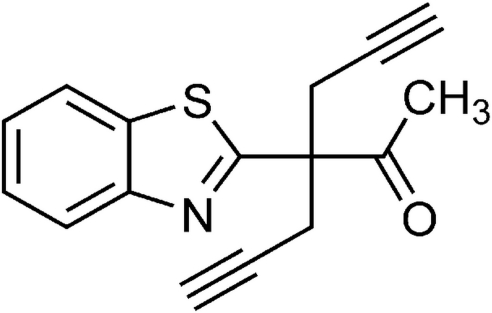


## Experimental

### 

#### Crystal data

C_16_H_13_NOS
*M* *_r_* = 267.34
Monoclinic, 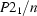

*a* = 7.7913 (1) Å
*b* = 30.2051 (6) Å
*c* = 12.4437 (2) Å
β = 106.161 (1)°
*V* = 2812.74 (8) Å^3^
*Z* = 8
Mo *K*α radiation
μ = 0.22 mm^−1^
*T* = 298 K
0.36 × 0.30 × 0.20 mm

#### Data collection

Bruker X8 APEXII CCD area-detector diffractometer
45783 measured reflections
8407 independent reflections
5449 reflections with *I* > 2σ(*I*)
*R*_int_ = 0.052

#### Refinement

*R*[*F* ^2^ > 2σ(*F* ^2^)] = 0.045
*wR*(*F* ^2^) = 0.128
*S* = 1.01
8407 reflections
391 parameters
H atoms treated by a mixture of independent and constrained refinement
Δρ_max_ = 0.31 e Å^−3^
Δρ_min_ = −0.19 e Å^−3^

### 

Data collection: *APEX2* (Bruker, 2005 ▶); cell refinement: *SAINT* (Bruker, 2005 ▶); data reduction: *SAINT*; program(s) used to solve structure: *SHELXS97* (Sheldrick, 2008 ▶); program(s) used to refine structure: *SHELXL97* (Sheldrick, 2008 ▶); molecular graphics: *ORTEP-3 for Windows* (Farrugia, 1997 ▶) and *PLATON* (Spek, 2009 ▶); software used to prepare material for publication: *WinGX* (Farrugia, 1999 ▶).

## Supplementary Material

Crystal structure: contains datablocks I, global. DOI: 10.1107/S1600536810009293/im2184sup1.cif

Structure factors: contains datablocks I. DOI: 10.1107/S1600536810009293/im2184Isup2.hkl

Additional supplementary materials:  crystallographic information; 3D view; checkCIF report

## Figures and Tables

**Table 1:** Hydrogen-bond geometry (Å, °)

*D*—H⋯*A*	*D*—H	H⋯*A*	*D*⋯*A*	*D*—H⋯*A*
C13—–H13⋯..O1^i^	0.93 (3)	2.52 (3)	3.409 (3)	161 (2)
C14—–H14*B*⋯..O2	0.97	2.39	3.302 (2)	155
C27—–H27*A*⋯..O1^ii^	0.97	2.55	3.409 (2)	147

Symmetry codes: (i) 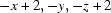; (ii) 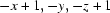.

**Fig. 1.**
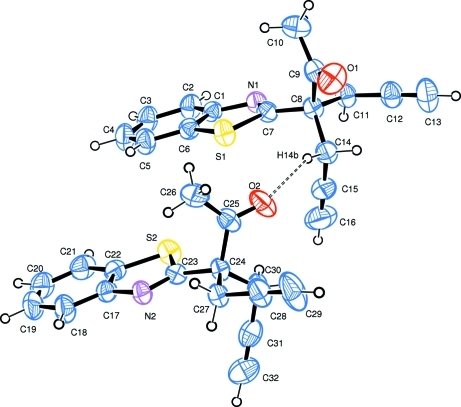
: Molecular structure of the title compound with the atom-labelling scheme. Displacement ellipsoids are drawn at the 50% probability level. H atoms are represented as small circles.

**Fig. 2.**
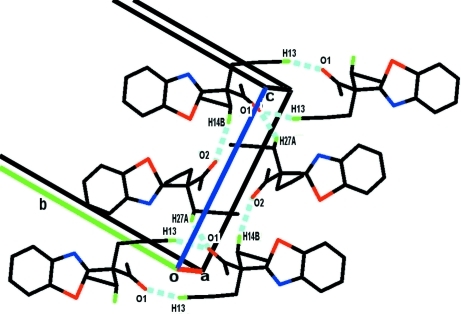
: Partial packing view showing the intermolecular C–H···O interactions linking the molecules into a 2-D array in the bc plane.

## supplementary crystallographic information

### Comment

Benzothiazoles possess therapeutic value, are synthetic intermediates in the preparation of medicinal compounds and find numerous applications in chemical industry (Bradshaw *et al.* 2002, Hutchinson *et al.* 2002, Delmas *et al.* 2002). Benzothiazole nucleus is associated with several pharmacological activities such as antitumoral (Repiĉ *et al.* 2001) and antimicrobial (Schwartz, *et al.* 1992). An alkylating reaction with propargyl bromide of 1-(benzothiazol-2-yl)propan-2-one (I) leading to the title compound 3-(benzothiazol-2-yl)-3-prop-2-ynyl-hex-5-yn-2-one (II) was performed employing either phase transfer catalysis or classical reaction conditions in acetone with potassium carbonate as a base.

The plot of the two molecules bulding the asymmetric unit is shown in Fig. 1. Each molecule consists of a benzothiazole moiety linked to dipropargylacetonyl group. The benzothiazole rings are essentially planar with maximum deviations of 0.005 (1) Å and 0.007 (2) Å from N1 and N2 respectively. The difference between the molecules is observed in the orientation of the two propargyl and acetonyl groups in each molecule (Spek, 2009). The dihedral angles in the first molecule, between S1-N1-C7 and C11-C12-C13, C14-C15-C16, O1-C9-C10 are 18 (4), 82 (9) and 87.5 (2)°, respectively. In the second molecule, we have 63 (8), 75 (4) and 88.8 (2)° respectively, between S2-N2-C23 and C27-C29-C28, C30-C31-C32 and O2-C25-C26. The two molecules within the asymmetric unit are linked by C—H···O hydrogen bonds building up a dimeric substructure. These dimers are further linked to each other by C—H···O hydrogen bonds forming in to 2-D array in the *bc* plane (Table 1, Fig. 2).

### Experimental

To a stirred solution containing 1 g (5.23 mmol) of 1-(benzothiazol-2-yl)propan -2-one (I), 1 g (7.43 mmol) of potassium carbonate and 20 mg of the catalyst benzyl triethylammonium bromide (BTBA) in 30 ml of dimethylformamide, was added in one portion 0.7 g (5.76 mmol) of propargyl bromide . The reaction mixture was stirred for 24 hours at room temperature. The mixture was extracted with dichloromethane (10 ml x 3). The organic layer was dried over Na_2_SO_4_ and evaporated to dryness *in vacuo* to get viscous liquid product, which was further precipitated after cooling. On recrystallization from ethanol brown single crystals of (II) (yield: 1.26 g; 90%; mp 110-112°C) were obtained.

### Refinement

H atoms were fixed geometrically and treated as riding with C—H = 0.96 Å for methyl groups and C—H = 0.93 Å for all other hydrogens with U_iso_(H) = 1.2 U_eq_(aromatic, methine ) or U_iso_(H) = 1.5 U_eq_(methyl). All other H atoms were located from difference Fourier maps and refined without any distance restraints.

### Crystal data

**Table:**

C_16_H_13_NOS	*Z* = 8
*M**_r_* = 267.34	*F*(000) = 1120
Monoclinic, *P*2_1_/*n*	*D*_x_ = 1.263 Mg m^−^^3^
Hall symbol: -p 2yn	Mo *K*α radiation, λ = 0.71073 Å
*a* = 7.7913 (1) Å	θ = 7.0–30.3°
*b* = 30.2051 (6) Å	µ = 0.22 mm^−^^1^
*c* = 12.4437 (2) Å	*T* = 298 K
β = 106.161 (1)°	Parallelepiped, clear pale yellow
*V* = 2812.74 (8) Å^3^	0.36 × 0.30 × 0.20 mm

### Data collection

**Table:**

Bruker X8 APEX CCD area-detector diffractometer	5449 reflections with *I* > 2σ(*I*)
Radiation source: fine-focus sealed tube	*R*_int_ = 0.052
graphite	θ_max_ = 30.3°, θ_min_ = 2.2°
φ and ω scans	*h* = −11→11
45783 measured reflections	*k* = −42→42
8407 independent reflections	*l* = −17→17

### Refinement

**Table:**

Refinement on *F*^2^	Primary atom site location: structure-invariant direct methods
Least-squares matrix: full	Secondary atom site location: difference Fourier map
*R*[*F*^2^ > 2σ(*F*^2^)] = 0.045	Hydrogen site location: inferred from neighbouring sites
*wR*(*F*^2^) = 0.128	H atoms treated by a mixture of independent and constrained refinement
*S* = 1.01	*w* = 1/[σ^2^(*F*_o_^2^) + (0.0611*P*)^2^ + 0.4268*P*] where *P* = (*F*_o_^2^ + 2*F*_c_^2^)/3
8407 reflections	(Δ/σ)_max_ = 0.001
391 parameters	Δρ_max_ = 0.31 e Å^−^^3^
0 restraints	Δρ_min_ = −0.19 e Å^−^^3^

### Special details

**Table:**

Geometry. All esds (except the esd in the dihedral angle between two l.s. planes) are estimated using the full covariance matrix. The cell esds are taken into account individually in the estimation of esds in distances, angles and torsion angles; correlations between esds in cell parameters are only used when they are defined by crystal symmetry. An approximate (isotropic) treatment of cell esds is used for estimating esds involving l.s. planes.
Refinement. Refinement of *F*^2^ against ALL reflections. The weighted *R*-factor wR and goodness of fit *S* are based on *F*^2^, conventional *R*-factors *R* are based on *F*, with *F* set to zero for negative *F*^2^. The threshold expression of *F*^2^ > σ(*F*^2^) is used only for calculating *R*-factors(gt) etc. and is not relevant to the choice of reflections for refinement. *R*-factors based on *F*^2^ are statistically about twice as large as those based on *F*, and *R*- factors based on ALL data will be even larger.

### Fractional atomic coordinates and isotropic or equivalent isotropic displacement parameters (Å^2^)

**Table:**

	*x*	*y*	*z*	*U*_iso_*/*U*_eq_	
O1	0.62822 (19)	0.02125 (4)	0.85422 (13)	0.0661 (4)	
S1	0.45815 (6)	0.126638 (14)	0.65398 (3)	0.04635 (12)	
N1	0.55435 (17)	0.17249 (4)	0.83678 (10)	0.0379 (3)	
C1	0.4413 (2)	0.19924 (5)	0.75663 (12)	0.0360 (3)	
C2	0.3957 (2)	0.24295 (6)	0.77253 (15)	0.0463 (4)	
H2	0.440 (2)	0.2551 (6)	0.8419 (16)	0.045 (5)*	
C3	0.2901 (2)	0.26604 (6)	0.68364 (16)	0.0514 (4)	
H3	0.260 (3)	0.2954 (7)	0.6956 (16)	0.059 (6)*	
C4	0.2271 (3)	0.24671 (7)	0.57885 (16)	0.0541 (5)	
H4	0.154 (3)	0.2633 (7)	0.5185 (17)	0.063 (6)*	
C5	0.2673 (2)	0.20358 (7)	0.56119 (15)	0.0496 (4)	
H5	0.222 (3)	0.1904 (7)	0.4940 (17)	0.062 (6)*	
C6	0.3754 (2)	0.17996 (5)	0.65053 (13)	0.0383 (3)	
C7	0.5758 (2)	0.13457 (5)	0.79517 (12)	0.0357 (3)	
C8	0.6974 (2)	0.09826 (5)	0.85737 (12)	0.0372 (3)	
C9	0.5878 (2)	0.05786 (5)	0.87635 (13)	0.0435 (4)	
C10	0.4340 (3)	0.06577 (7)	0.92442 (17)	0.0601 (5)	
H10A	0.4657	0.0883	0.9808	0.090*	
H10B	0.4062	0.0389	0.9571	0.090*	
H10C	0.3317	0.0752	0.8661	0.090*	
C11	0.8066 (2)	0.11589 (6)	0.97374 (13)	0.0442 (4)	
H11A	0.8691	0.1427	0.9639	0.053*	
H11B	0.7256	0.1232	1.0178	0.053*	
C12	0.9358 (3)	0.08305 (6)	1.03360 (14)	0.0516 (4)	
C13	1.0392 (4)	0.05574 (9)	1.07695 (19)	0.0747 (7)	
H13	1.122 (4)	0.0344 (9)	1.112 (2)	0.099 (9)*	
C14	0.8253 (2)	0.08345 (6)	0.78856 (14)	0.0453 (4)	
H14A	0.9067	0.0612	0.8303	0.054*	
H14B	0.7560	0.0701	0.7191	0.054*	
C15	0.9280 (3)	0.12022 (7)	0.76328 (16)	0.0543 (5)	
C16	1.0073 (3)	0.15030 (10)	0.7428 (2)	0.0793 (7)	
H16	1.067 (4)	0.1724 (8)	0.724 (2)	0.088 (8)*	
O2	0.6762 (2)	0.05561 (5)	0.52220 (10)	0.0639 (4)	
S2	0.55463 (6)	0.170295 (14)	0.37374 (3)	0.04545 (12)	
N2	0.49980 (18)	0.12720 (4)	0.18749 (10)	0.0388 (3)	
C17	0.4081 (2)	0.16709 (5)	0.16241 (13)	0.0391 (3)	
C18	0.3085 (3)	0.18000 (7)	0.05589 (16)	0.0563 (5)	
H18	0.299 (3)	0.1604 (7)	−0.0094 (19)	0.074 (7)*	
C19	0.2278 (3)	0.22127 (8)	0.04356 (19)	0.0658 (6)	
H19	0.162 (3)	0.2295 (7)	−0.0307 (18)	0.072 (6)*	
C20	0.2450 (3)	0.24907 (7)	0.1344 (2)	0.0621 (5)	
H20	0.183 (3)	0.2782 (7)	0.1227 (18)	0.072 (6)*	
C21	0.3403 (3)	0.23676 (6)	0.24036 (18)	0.0540 (5)	
H21	0.353 (3)	0.2552 (7)	0.3008 (17)	0.068 (6)*	
C22	0.4227 (2)	0.19521 (5)	0.25372 (13)	0.0395 (3)	
C23	0.5807 (2)	0.12458 (5)	0.29265 (12)	0.0338 (3)	
C24	0.6913 (2)	0.08534 (5)	0.34795 (12)	0.0356 (3)	
C25	0.5961 (2)	0.06227 (5)	0.42620 (13)	0.0434 (4)	
C26	0.4072 (3)	0.04820 (7)	0.37852 (18)	0.0634 (5)	
H26A	0.3602	0.0613	0.3059	0.095*	
H26B	0.3376	0.0577	0.4269	0.095*	
H26C	0.4018	0.0165	0.3719	0.095*	
C27	0.7065 (2)	0.05229 (5)	0.25588 (12)	0.0395 (3)	
H27A	0.5876	0.0431	0.2137	0.047*	
H27B	0.7615	0.0671	0.2047	0.047*	
C28	0.8117 (3)	0.01322 (7)	0.30197 (15)	0.0583 (5)	
C29	0.8994 (5)	−0.01727 (10)	0.3395 (2)	0.1035 (11)	
H29	0.970 (4)	−0.0425 (11)	0.363 (3)	0.128 (11)*	
C30	0.8784 (2)	0.10077 (6)	0.41712 (14)	0.0499 (4)	
H30A	0.8661	0.1203	0.4765	0.060*	
H30B	0.9477	0.0752	0.4515	0.060*	
C31	0.9732 (2)	0.12394 (6)	0.34835 (17)	0.0527 (4)	
C32	1.0447 (3)	0.14150 (8)	0.2886 (2)	0.0713 (6)	
H32	1.099 (4)	0.1568 (10)	0.237 (3)	0.123 (11)*	

### Atomic displacement parameters (Å^2^)

**Table:**

	*U*^11^	*U*^22^	*U*^33^	*U*^12^	*U*^13^	*U*^23^
O1	0.0675 (9)	0.0358 (7)	0.0939 (11)	−0.0001 (6)	0.0208 (8)	−0.0075 (7)
S1	0.0574 (3)	0.0398 (2)	0.0367 (2)	0.00420 (18)	0.00457 (18)	−0.00849 (16)
N1	0.0421 (7)	0.0375 (7)	0.0339 (6)	0.0034 (5)	0.0102 (5)	−0.0030 (5)
C1	0.0343 (8)	0.0377 (8)	0.0362 (7)	0.0020 (6)	0.0103 (6)	−0.0021 (6)
C2	0.0481 (10)	0.0430 (9)	0.0454 (9)	0.0083 (7)	0.0092 (8)	−0.0074 (7)
C3	0.0486 (10)	0.0437 (10)	0.0583 (11)	0.0160 (8)	0.0089 (8)	−0.0015 (8)
C4	0.0479 (10)	0.0568 (12)	0.0509 (10)	0.0143 (9)	0.0029 (8)	0.0066 (8)
C5	0.0469 (10)	0.0575 (11)	0.0387 (9)	0.0050 (8)	0.0022 (8)	−0.0022 (8)
C6	0.0355 (8)	0.0411 (8)	0.0380 (7)	−0.0008 (6)	0.0095 (6)	−0.0030 (6)
C7	0.0399 (8)	0.0350 (8)	0.0320 (7)	−0.0012 (6)	0.0097 (6)	−0.0025 (6)
C8	0.0434 (8)	0.0330 (8)	0.0349 (7)	0.0029 (6)	0.0102 (6)	−0.0030 (6)
C9	0.0509 (10)	0.0360 (9)	0.0395 (8)	−0.0005 (7)	0.0059 (7)	0.0001 (6)
C10	0.0698 (13)	0.0564 (12)	0.0613 (11)	−0.0093 (10)	0.0302 (10)	0.0011 (9)
C11	0.0499 (10)	0.0423 (9)	0.0370 (8)	0.0031 (7)	0.0065 (7)	−0.0044 (6)
C12	0.0552 (11)	0.0539 (11)	0.0412 (9)	0.0043 (9)	0.0060 (8)	−0.0048 (8)
C13	0.0797 (16)	0.0767 (16)	0.0555 (12)	0.0263 (13)	−0.0016 (11)	0.0013 (11)
C14	0.0508 (10)	0.0452 (9)	0.0405 (8)	0.0097 (7)	0.0139 (7)	−0.0015 (7)
C15	0.0501 (10)	0.0620 (12)	0.0560 (10)	0.0085 (9)	0.0234 (9)	0.0035 (9)
C16	0.0670 (15)	0.0808 (18)	0.1034 (19)	−0.0018 (13)	0.0460 (15)	0.0091 (14)
O2	0.0870 (10)	0.0675 (9)	0.0372 (6)	0.0073 (8)	0.0172 (7)	0.0111 (6)
S2	0.0591 (3)	0.0369 (2)	0.0393 (2)	0.00400 (18)	0.01197 (19)	−0.00679 (16)
N2	0.0446 (7)	0.0345 (7)	0.0348 (6)	0.0052 (6)	0.0072 (6)	0.0006 (5)
C17	0.0383 (8)	0.0361 (8)	0.0431 (8)	0.0041 (6)	0.0117 (7)	0.0047 (6)
C18	0.0616 (12)	0.0554 (11)	0.0476 (10)	0.0185 (9)	0.0080 (9)	0.0062 (8)
C19	0.0651 (13)	0.0678 (14)	0.0622 (12)	0.0292 (11)	0.0138 (11)	0.0211 (11)
C20	0.0615 (12)	0.0481 (11)	0.0815 (15)	0.0242 (10)	0.0280 (11)	0.0162 (10)
C21	0.0571 (11)	0.0391 (10)	0.0713 (13)	0.0095 (8)	0.0272 (10)	0.0009 (9)
C22	0.0392 (8)	0.0334 (8)	0.0488 (9)	0.0008 (6)	0.0170 (7)	0.0020 (6)
C23	0.0384 (8)	0.0288 (7)	0.0345 (7)	−0.0020 (6)	0.0107 (6)	−0.0022 (6)
C24	0.0416 (8)	0.0313 (7)	0.0311 (7)	0.0030 (6)	0.0056 (6)	0.0003 (5)
C25	0.0624 (11)	0.0311 (8)	0.0387 (8)	0.0070 (7)	0.0171 (8)	0.0026 (6)
C26	0.0670 (13)	0.0606 (13)	0.0681 (13)	−0.0082 (10)	0.0277 (11)	0.0132 (10)
C27	0.0472 (9)	0.0354 (8)	0.0338 (7)	0.0058 (7)	0.0080 (7)	−0.0016 (6)
C28	0.0786 (14)	0.0538 (11)	0.0425 (9)	0.0245 (10)	0.0166 (9)	0.0008 (8)
C29	0.158 (3)	0.093 (2)	0.0589 (14)	0.080 (2)	0.0293 (16)	0.0151 (13)
C30	0.0471 (10)	0.0504 (10)	0.0426 (9)	0.0029 (8)	−0.0030 (7)	−0.0021 (7)
C31	0.0404 (9)	0.0481 (10)	0.0633 (11)	−0.0031 (8)	0.0041 (8)	−0.0116 (9)
C32	0.0557 (13)	0.0712 (15)	0.0896 (17)	−0.0181 (11)	0.0245 (12)	−0.0116 (13)

### Geometric parameters (Å, °)

**Table:**

O1—C9	1.203 (2)	O2—C25	1.201 (2)
S1—C6	1.7308 (17)	S2—C22	1.7319 (17)
S1—C7	1.7573 (16)	S2—C23	1.7549 (15)
N1—C7	1.2869 (19)	N2—C23	1.2873 (19)
N1—C1	1.3909 (19)	N2—C17	1.3912 (19)
C1—C2	1.396 (2)	C17—C18	1.393 (2)
C1—C6	1.403 (2)	C17—C22	1.398 (2)
C2—C3	1.371 (2)	C18—C19	1.385 (3)
C2—H2	0.912 (18)	C18—H18	0.99 (2)
C3—C4	1.388 (3)	C19—C20	1.384 (3)
C3—H3	0.94 (2)	C19—H19	0.96 (2)
C4—C5	1.372 (3)	C20—C21	1.372 (3)
C4—H4	0.95 (2)	C20—H20	1.00 (2)
C5—C6	1.390 (2)	C21—C22	1.398 (2)
C5—H5	0.91 (2)	C21—H21	0.92 (2)
C7—C8	1.514 (2)	C23—C24	1.514 (2)
C8—C9	1.545 (2)	C24—C30	1.544 (2)
C8—C14	1.549 (2)	C24—C25	1.544 (2)
C8—C11	1.555 (2)	C24—C27	1.549 (2)
C9—C10	1.500 (3)	C25—C26	1.488 (3)
C10—H10A	0.9600	C26—H26A	0.9600
C10—H10B	0.9600	C26—H26B	0.9600
C10—H10C	0.9600	C26—H26C	0.9600
C11—C12	1.461 (2)	C27—C28	1.460 (2)
C11—H11A	0.9700	C27—H27A	0.9700
C11—H11B	0.9700	C27—H27B	0.9700
C12—C13	1.174 (3)	C28—C29	1.164 (3)
C13—H13	0.93 (3)	C29—H29	0.94 (3)
C14—C15	1.454 (3)	C30—C31	1.456 (3)
C14—H14A	0.9700	C30—H30A	0.9700
C14—H14B	0.9700	C30—H30B	0.9700
C15—C16	1.166 (3)	C31—C32	1.173 (3)
C16—H16	0.88 (3)	C32—H32	0.98 (3)
			
C6—S1—C7	89.14 (7)	C22—S2—C23	88.96 (7)
C7—N1—C1	110.83 (13)	C23—N2—C17	110.90 (13)
N1—C1—C2	125.68 (14)	N2—C17—C18	124.86 (15)
N1—C1—C6	115.25 (14)	N2—C17—C22	115.06 (14)
C2—C1—C6	119.03 (15)	C18—C17—C22	120.08 (15)
C3—C2—C1	119.07 (16)	C19—C18—C17	118.23 (19)
C3—C2—H2	122.9 (11)	C19—C18—H18	121.3 (13)
C1—C2—H2	118.0 (11)	C17—C18—H18	120.4 (12)
C2—C3—C4	121.33 (17)	C20—C19—C18	121.16 (19)
C2—C3—H3	118.2 (12)	C20—C19—H19	122.3 (13)
C4—C3—H3	120.5 (12)	C18—C19—H19	116.5 (13)
C5—C4—C3	120.89 (17)	C21—C20—C19	121.61 (18)
C5—C4—H4	119.2 (12)	C21—C20—H20	119.4 (13)
C3—C4—H4	119.9 (12)	C19—C20—H20	119.0 (13)
C4—C5—C6	118.28 (17)	C20—C21—C22	117.70 (19)
C4—C5—H5	121.3 (13)	C20—C21—H21	121.9 (14)
C6—C5—H5	120.4 (13)	C22—C21—H21	120.4 (14)
C5—C6—C1	121.39 (15)	C17—C22—C21	121.20 (16)
C5—C6—S1	129.48 (13)	C17—C22—S2	109.35 (11)
C1—C6—S1	109.07 (12)	C21—C22—S2	129.44 (14)
N1—C7—C8	124.89 (13)	N2—C23—C24	124.47 (13)
N1—C7—S1	115.70 (12)	N2—C23—S2	115.73 (11)
C8—C7—S1	119.37 (11)	C24—C23—S2	119.80 (10)
C7—C8—C9	110.88 (13)	C23—C24—C30	110.29 (13)
C7—C8—C14	109.50 (12)	C23—C24—C25	109.11 (12)
C9—C8—C14	108.92 (13)	C30—C24—C25	109.21 (13)
C7—C8—C11	109.28 (12)	C23—C24—C27	108.63 (12)
C9—C8—C11	108.09 (13)	C30—C24—C27	110.63 (13)
C14—C8—C11	110.16 (13)	C25—C24—C27	108.93 (12)
O1—C9—C10	121.82 (17)	O2—C25—C26	122.09 (17)
O1—C9—C8	120.00 (16)	O2—C25—C24	119.67 (17)
C10—C9—C8	118.18 (14)	C26—C25—C24	118.22 (14)
C9—C10—H10A	109.5	C25—C26—H26A	109.5
C9—C10—H10B	109.5	C25—C26—H26B	109.5
H10A—C10—H10B	109.5	H26A—C26—H26B	109.5
C9—C10—H10C	109.5	C25—C26—H26C	109.5
H10A—C10—H10C	109.5	H26A—C26—H26C	109.5
H10B—C10—H10C	109.5	H26B—C26—H26C	109.5
C12—C11—C8	111.27 (14)	C28—C27—C24	112.37 (13)
C12—C11—H11A	109.4	C28—C27—H27A	109.1
C8—C11—H11A	109.4	C24—C27—H27A	109.1
C12—C11—H11B	109.4	C28—C27—H27B	109.1
C8—C11—H11B	109.4	C24—C27—H27B	109.1
H11A—C11—H11B	108.0	H27A—C27—H27B	107.9
C13—C12—C11	176.8 (2)	C29—C28—C27	178.3 (3)
C12—C13—H13	179.2 (18)	C28—C29—H29	174.5 (19)
C15—C14—C8	112.10 (14)	C31—C30—C24	111.84 (14)
C15—C14—H14A	109.2	C31—C30—H30A	109.2
C8—C14—H14A	109.2	C24—C30—H30A	109.2
C15—C14—H14B	109.2	C31—C30—H30B	109.2
C8—C14—H14B	109.2	C24—C30—H30B	109.2
H14A—C14—H14B	107.9	H30A—C30—H30B	107.9
C16—C15—C14	178.6 (2)	C32—C31—C30	176.8 (2)
C15—C16—H16	177.2 (18)	C31—C32—H32	177.4 (19)
			
C7—N1—C1—C2	−177.23 (16)	C23—N2—C17—C18	179.57 (17)
C7—N1—C1—C6	0.27 (19)	C23—N2—C17—C22	0.14 (19)
N1—C1—C2—C3	176.44 (16)	N2—C17—C18—C19	−178.52 (18)
C6—C1—C2—C3	−1.0 (3)	C22—C17—C18—C19	0.9 (3)
C1—C2—C3—C4	0.4 (3)	C17—C18—C19—C20	−0.1 (3)
C2—C3—C4—C5	0.7 (3)	C18—C19—C20—C21	−0.9 (4)
C3—C4—C5—C6	−1.1 (3)	C19—C20—C21—C22	1.0 (3)
C4—C5—C6—C1	0.5 (3)	N2—C17—C22—C21	178.71 (15)
C4—C5—C6—S1	−176.61 (15)	C18—C17—C22—C21	−0.7 (3)
N1—C1—C6—C5	−177.15 (15)	N2—C17—C22—S2	−0.31 (17)
C2—C1—C6—C5	0.5 (2)	C18—C17—C22—S2	−179.77 (14)
N1—C1—C6—S1	0.50 (17)	C20—C21—C22—C17	−0.2 (3)
C2—C1—C6—S1	178.18 (13)	C20—C21—C22—S2	178.59 (15)
C7—S1—C6—C5	176.60 (17)	C23—S2—C22—C17	0.29 (12)
C7—S1—C6—C1	−0.80 (12)	C23—S2—C22—C21	−178.63 (16)
C1—N1—C7—C8	176.84 (13)	C17—N2—C23—C24	179.44 (13)
C1—N1—C7—S1	−0.94 (17)	C17—N2—C23—S2	0.10 (17)
C6—S1—C7—N1	1.05 (13)	C22—S2—C23—N2	−0.24 (13)
C6—S1—C7—C8	−176.85 (12)	C22—S2—C23—C24	−179.61 (12)
N1—C7—C8—C9	112.90 (17)	N2—C23—C24—C30	127.53 (16)
S1—C7—C8—C9	−69.40 (15)	S2—C23—C24—C30	−53.15 (16)
N1—C7—C8—C14	−126.88 (16)	N2—C23—C24—C25	−112.51 (16)
S1—C7—C8—C14	50.82 (16)	S2—C23—C24—C25	66.81 (15)
N1—C7—C8—C11	−6.1 (2)	N2—C23—C24—C27	6.1 (2)
S1—C7—C8—C11	171.56 (11)	S2—C23—C24—C27	−174.56 (11)
C7—C8—C9—O1	131.63 (17)	C23—C24—C25—O2	−126.98 (16)
C14—C8—C9—O1	11.1 (2)	C30—C24—C25—O2	−6.3 (2)
C11—C8—C9—O1	−108.61 (18)	C27—C24—C25—O2	114.58 (17)
C7—C8—C9—C10	−49.44 (19)	C23—C24—C25—C26	54.16 (19)
C14—C8—C9—C10	−170.00 (15)	C30—C24—C25—C26	174.79 (15)
C11—C8—C9—C10	70.32 (18)	C27—C24—C25—C26	−64.29 (19)
C7—C8—C11—C12	−175.71 (14)	C23—C24—C27—C28	179.97 (15)
C9—C8—C11—C12	63.53 (18)	C30—C24—C27—C28	58.76 (19)
C14—C8—C11—C12	−55.37 (18)	C25—C24—C27—C28	−61.29 (19)
C8—C11—C12—C13	22 (4)	C24—C27—C28—C29	−68 (7)
C7—C8—C14—C15	56.19 (18)	C23—C24—C30—C31	−58.50 (18)
C9—C8—C14—C15	177.59 (14)	C25—C24—C30—C31	−178.41 (14)
C11—C8—C14—C15	−64.02 (18)	C27—C24—C30—C31	61.71 (18)
C8—C14—C15—C16	−35 (10)	C24—C30—C31—C32	−26 (4)

### Hydrogen-bond geometry (Å, °)

**Table:**

*D*—H···*A*	*D*—H	H···*A*	*D*···*A*	*D*—H···*A*
C13—–H13···..O1^i^	0.93 (3)	2.52 (3)	3.409 (3)	161 (2)
C14—–H14B···..O2	0.97	2.39	3.302 (2)	155
C27—–H27A···..O1^ii^	0.97	2.55	3.409 (2)	147

Symmetry codes: (i) −*x*+2, −*y*, −*z*+2; (ii) −*x*+1, −*y*, −*z*+1.
